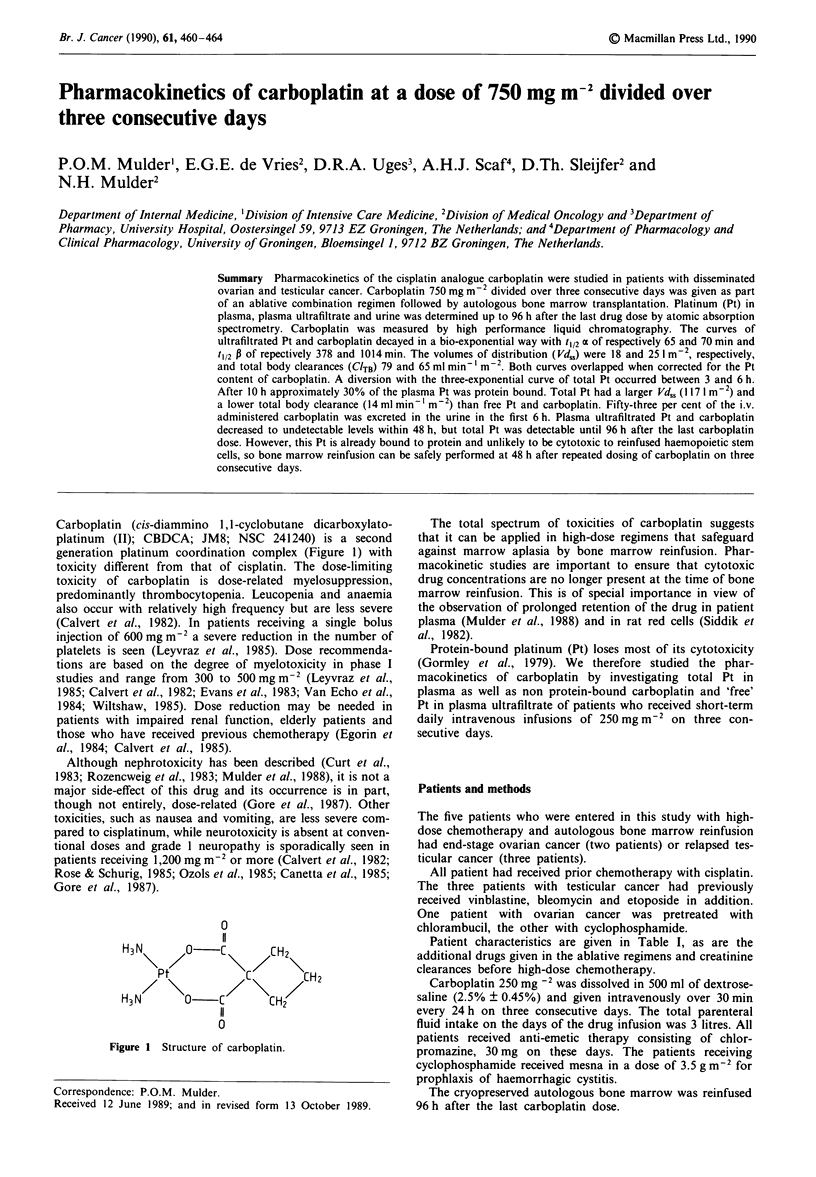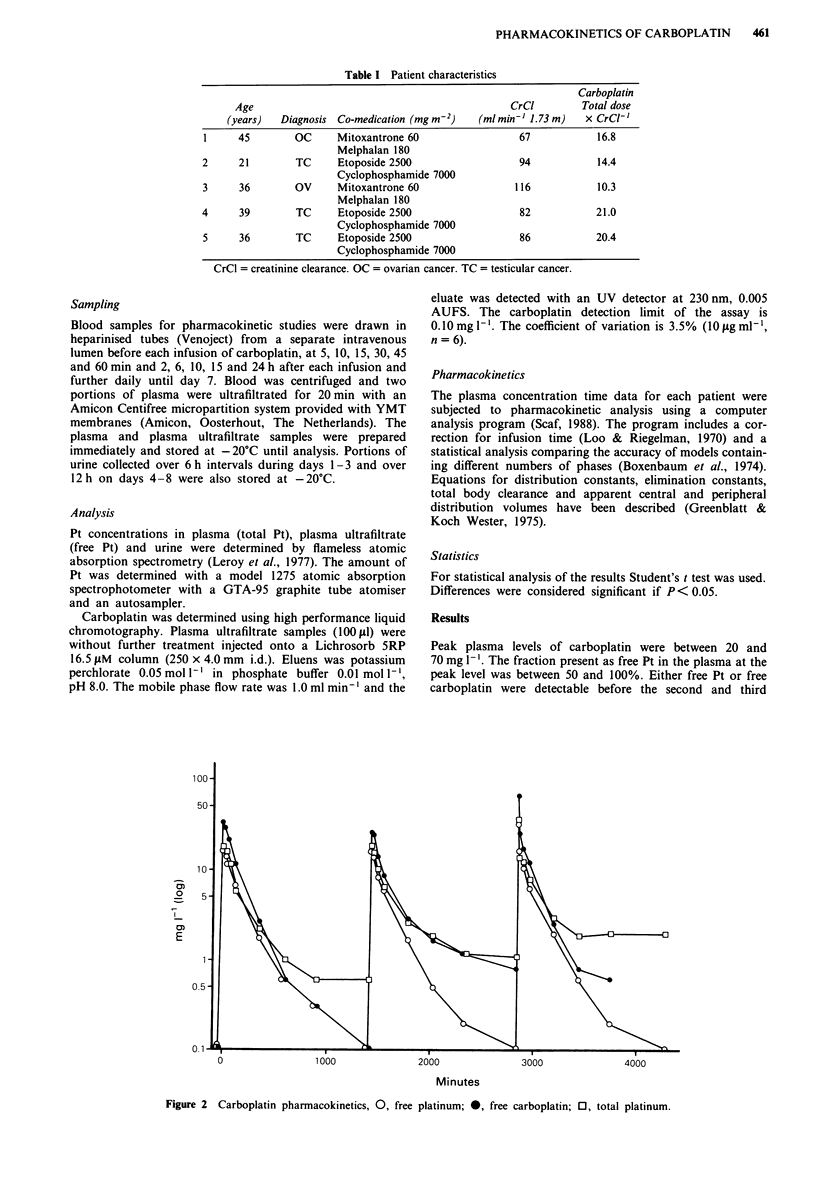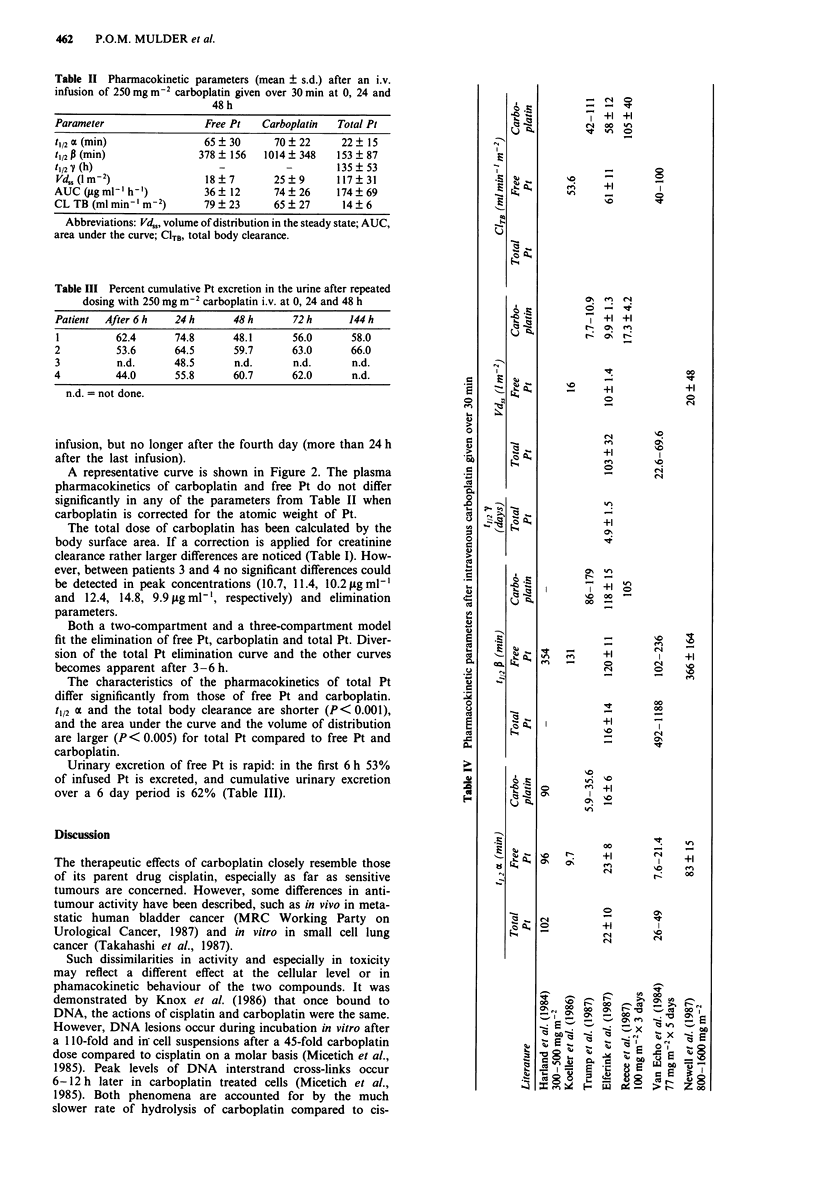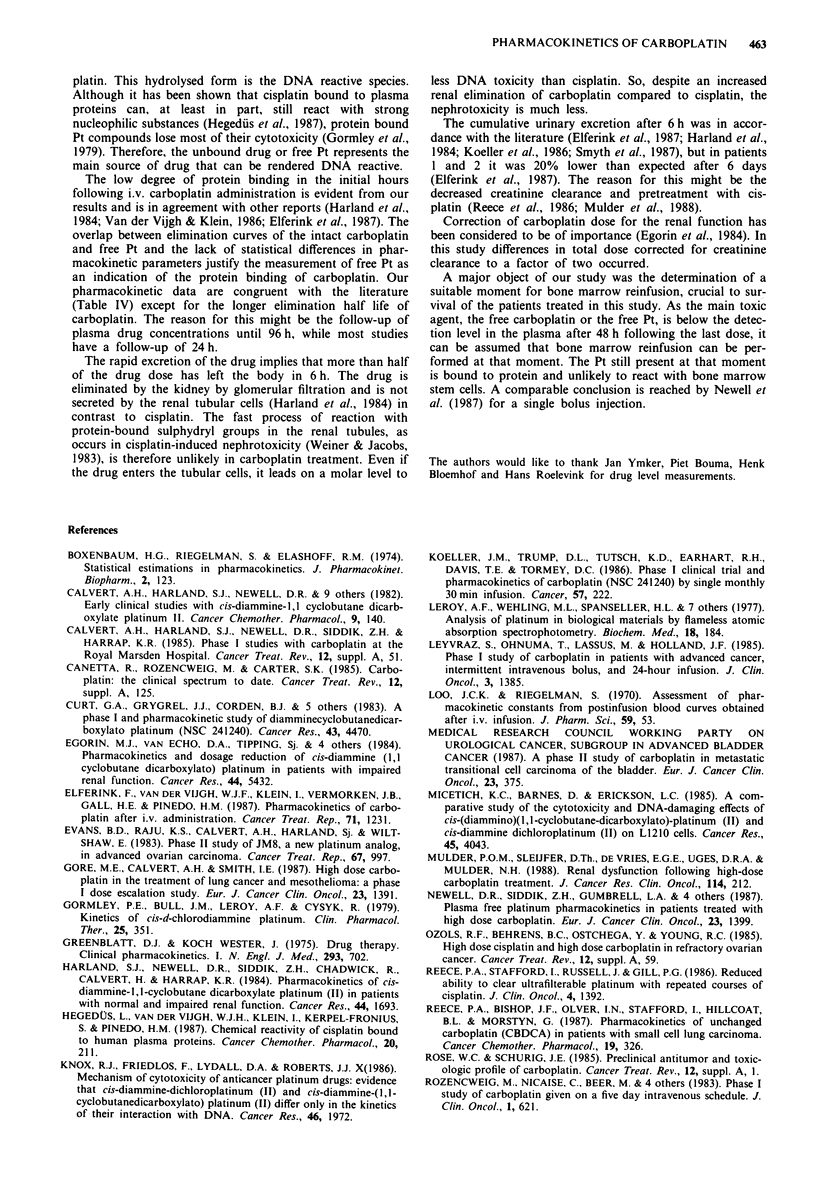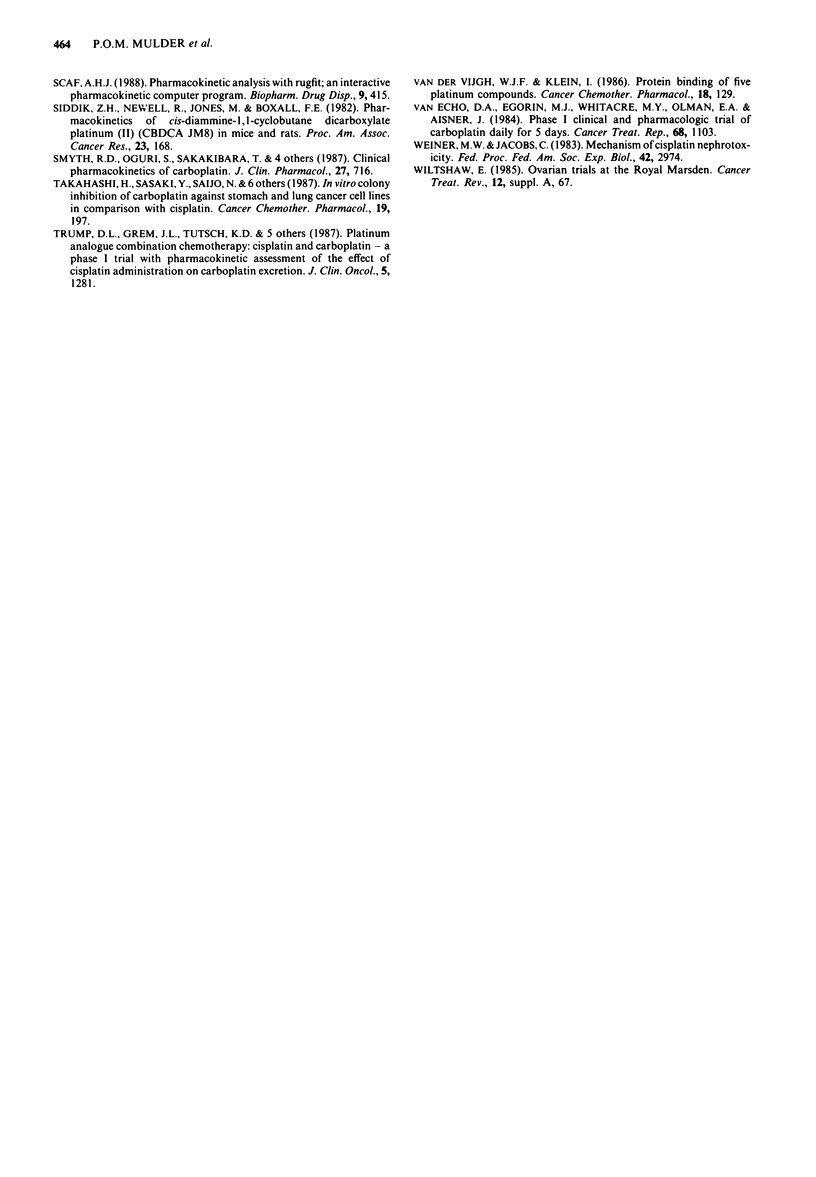# Pharmacokinetics of carboplatin at a dose of 750 mg m-2 divided over three consecutive days.

**DOI:** 10.1038/bjc.1990.101

**Published:** 1990-03

**Authors:** P. O. Mulder, E. G. de Vries, D. R. Uges, A. H. Scaf, D. T. Sleijfer, N. H. Mulder

**Affiliations:** Department of Internal Medicine, University Hospital, Groningen, The Netherlands.

## Abstract

Pharmacokinetics of the cisplatin analogue carboplatin were studied in patients with disseminated ovarian and testicular cancer. Carboplatin 750 mg m-2 divided over three consecutive days was given as part of an ablative combination regimen followed by autologous bone marrow transplantation. Platinum (Pt) in plasma, plasma ultrafiltrate and urine was determined up to 96 h after the last drug dose by atomic absorption spectrometry. Carboplatin was measured by high performance liquid chromatography. The curves of ultrafiltrated Pt and carboplatin decayed in a bio-exponential way with t1/2 alpha of respectively 65 and 70 min and t1/2 beta of respectively 378 and 1014 min. The volumes of distribution (Vdss) were 18 and 25 l m-2, respectively, and total body clearances (ClTB) 79 and 65 ml min-1 m-2. Both curves overlapped when corrected for the Pt content of carboplatin. A diversion with the three-exponential curve of total Pt occurred between 3 and 6 h. After 10 h approximately 30% of the plasma Pt was protein bound. Total Pt had a larger Vdss (117 l m-2) and a lower total body clearance (14 ml min-1 m-2) than free Pt and carboplatin. Fifty-three per cent of the i.v. administered carboplatin was excreted in the urine in the first 6 h. Plasma ultrafiltrated Pt and carboplatin decreased to undetectable levels within 48 h, but total Pt was detectable until 96 h after the last carboplatin dose. However, this Pt is already bound to protein and unlikely to be cytotoxic to reinfused haemopoietic stem cells, so bone marrow reinfusion can be safely performed at 48 h after repeated dosing of carboplatin on three consecutive days.


*Br. J. Cancer* (1990), **61**, 460–464 © Macmillan Press Ltd., 1990

### Pharmacokinetics of carboplatin at a dose of 750 mg m$^{-2}$ divided over three consecutive days

P.O.M. Mulder[1], E.G.E. de Vries[2], D.R.A. Uges[3], A.H.J. Scaf[4], D.Th. Sleijfer[2] and N.H. Mulder[2]

*Department of Internal Medicine, [1]Division of Intensive Care Medicine, [2]Division of Medical Oncology and [3]Department of Pharmacy, University Hospital, Oostersingel 59, 9713 EZ Groningen, The Netherlands; and [4]Department of Pharmacology and Clinical Pharmacology, University of Groningen, Bloemsingel 1, 9712 BZ Groningen, The Netherlands.*

**Summary** Pharmacokinetics of the cisplatin analogue carboplatin were studied in patients with disseminated ovarian and testicular cancer. Carboplatin 750 mg m$^{-2}$ divided over three consecutive days was given as part of an ablative combination regimen followed by autologous bone marrow transplantation. Platinum (Pt) in plasma, plasma ultrafiltrate and urine was determined up to 96 h after the last drug dose by atomic absorption spectrometry. Carboplatin was measured by high performance liquid chromatography. The curves of ultrafiltrated Pt and carboplatin decayed in a bio-exponential way with $t_{1/2}\ \alpha$ of respectively 65 and 70 min and $t_{1/2}\ \beta$ of repectively 378 and 1014 min. The volumes of distribution ($Vd_{ss}$) were 18 and 25 l m$^{-2}$, respectively, and total body clearances ($Cl_{TB}$) 79 and 65 ml min$^{-1}$ m$^{-2}$. Both curves overlapped when corrected for the Pt content of carboplatin. A diversion with the three-exponential curve of total Pt occurred between 3 and 6 h. After 10 h approximately 30% of the plasma Pt was protein bound. Total Pt had a larger $Vd_{ss}$ (117 l m$^{-2}$) and a lower total body clearance (14 ml min$^{-1}$ m$^{-2}$) than free Pt and carboplatin. Fifty-three per cent of the i.v. administered carboplatin was excreted in the urine in the first 6 h. Plasma ultrafiltrated Pt and carboplatin decreased to undetectable levels within 48 h, but total Pt was detectable until 96 h after the last carboplatin dose. However, this Pt is already bound to protein and unlikely to be cytotoxic to reinfused haemopoietic stem cells, so bone marrow reinfusion can be safely performed at 48 h after repeated dosing of carboplatin on three consecutive days.

Carboplatin (*cis*-diammino 1,1-cyclobutane dicarboxylato-platinum (II); CBDCA; JM8; NSC 241240) is a second generation platinum coordination complex (Figure 1) with toxicity different from that of cisplatin. The dose-limiting toxicity of carboplatin is dose-related myelosuppression, predominantly thrombocytopenia. Leucopenia and anaemia also occur with relatively high frequency but are less severe (Calvert *et al.*, 1982). In patients receiving a single bolus injection of 600 mg m$^{-2}$ a severe reduction in the number of platelets is seen (Leyvraz *et al.*, 1985). Dose recommendations are based on the degree of myelotoxicity in phase I studies and range from 300 to 500 mg m$^{-2}$ (Leyvraz *et al.*, 1985; Calvert *et al.*, 1982; Evans *et al.*, 1983; Van Echo *et al.*, 1984; Wiltshaw, 1985). Dose reduction may be needed in patients with impaired renal function, elderly patients and those who have received previous chemotherapy (Egorin *et al.*, 1984; Calvert *et al.*, 1985).

Although nephrotoxicity has been described (Curt *et al.*, 1983; Rozencweig *et al.*, 1983; Mulder *et al.*, 1988), it is not a major side-effect of this drug and its occurrence is in part, though not entirely, dose-related (Gore *et al.*, 1987). Other toxicities, such as nausea and vomiting, are less severe compared to cisplatinum, while neurotoxicity is absent at conventional doses and grade 1 neuropathy is sporadically seen in patients receiving 1,200 mg m$^{-2}$ or more (Calvert *et al.*, 1982; Rose & Schurig, 1985; Ozols *et al.*, 1985; Canetta *et al.*, 1985; Gore *et al.*, 1987).

**Figure 1** Structure of carboplatin.

Correspondence: P.O.M. Mulder.
Received 12 June 1989; and in revised form 13 October 1989.

The total spectrum of toxicities of carboplatin suggests that it can be applied in high-dose regimens that safeguard against marrow aplasia by bone marrow reinfusion. Pharmacokinetic studies are important to ensure that cytotoxic drug concentrations are no longer present at the time of bone marrow reinfusion. This is of special importance in view of the observation of prolonged retention of the drug in patient plasma (Mulder *et al.*, 1988) and in rat red cells (Siddik *et al.*, 1982).

Protein-bound platinum (Pt) loses most of its cytotoxicity (Gormley *et al.*, 1979). We therefore studied the pharmacokinetics of carboplatin by investigating total Pt in plasma as well as non protein-bound carboplatin and 'free' Pt in plasma ultrafiltrate of patients who received short-term daily intravenous infusions of 250 mg m$^{-2}$ on three consecutive days.

### Patients and methods

The five patients who were entered in this study with high-dose chemotherapy and autologous bone marrow reinfusion had end-stage ovarian cancer (two patients) or relapsed testicular cancer (three patients).

All patient had received prior chemotherapy with cisplatin. The three patients with testicular cancer had previously received vinblastine, bleomycin and etoposide in addition. One patient with ovarian cancer was pretreated with chlorambucil, the other with cyclophosphamide.

Patient characteristics are given in Table I, as are the additional drugs given in the ablative regimens and creatinine clearances before high-dose chemotherapy.

Carboplatin 250 mg $^{-2}$ was dissolved in 500 ml of dextrose-saline (2.5% ± 0.45%) and given intravenously over 30 min every 24 h on three consecutive days. The total parenteral fluid intake on the days of the drug infusion was 3 litres. All patients received anti-emetic therapy consisting of chlorpromazine, 30 mg on these days. The patients receiving cyclophosphamide received mesna in a dose of 3.5 g m$^{-2}$ for prophlaxis of haemorrhagic cystitis.

The cryopreserved autologous bone marrow was reinfused 96 h after the last carboplatin dose.

**Table I** Patient characteristics

| | *Age (years)* | *Diagnosis* | *Co-medication (mg m$^{-2}$)* | *CrCl (ml min$^{-1}$ 1.73 m)* | *Carboplatin Total dose × CrCl$^{-1}$* |
|---|---|---|---|---|---|
| 1 | 45 | OC | Mitoxantrone 60<br>Melphalan 180 | 67 | 16.8 |
| 2 | 21 | TC | Etoposide 2500<br>Cyclophosphamide 7000 | 94 | 14.4 |
| 3 | 36 | OV | Mitoxantrone 60<br>Melphalan 180 | 116 | 10.3 |
| 4 | 39 | TC | Etoposide 2500<br>Cyclophosphamide 7000 | 82 | 21.0 |
| 5 | 36 | TC | Etoposide 2500<br>Cyclophosphamide 7000 | 86 | 20.4 |

CrCl = creatinine clearance. OC = ovarian cancer. TC = testicular cancer.

*Sampling*

Blood samples for pharmacokinetic studies were drawn in heparinised tubes (Venoject) from a separate intravenous lumen before each infusion of carboplatin, at 5, 10, 15, 30, 45 and 60 min and 2, 6, 10, 15 and 24 h after each infusion and further daily until day 7. Blood was centrifuged and two portions of plasma were ultrafiltrated for 20 min with an Amicon Centifree micropartition system provided with YMT membranes (Amicon, Oosterhout, The Netherlands). The plasma and plasma ultrafiltrate samples were prepared immediately and stored at −20°C until analysis. Portions of urine collected over 6 h intervals during days 1–3 and over 12 h on days 4–8 were also stored at −20°C.

*Analysis*

Pt concentrations in plasma (total Pt), plasma ultrafiltrate (free Pt) and urine were determined by flameless atomic absorption spectrometry (Leroy *et al.*, 1977). The amount of Pt was determined with a model 1275 atomic absorption spectrophotometer with a GTA-95 graphite tube atomiser and an autosampler.

Carboplatin was determined using high performance liquid chromotography. Plasma ultrafiltrate samples (100 µl) were without further treatment injected onto a Lichrosorb 5RP 16.5 µM column (250 × 4.0 mm i.d.). Eluens was potassium perchlorate 0.05 mol l$^{-1}$ in phosphate buffer 0.01 mol l$^{-1}$, pH 8.0. The mobile phase flow rate was 1.0 ml min$^{-1}$ and the eluate was detected with an UV detector at 230 nm, 0.005 AUFS. The carboplatin detection limit of the assay is 0.10 mg l$^{-1}$. The coefficient of variation is 3.5% (10 µg ml$^{-1}$, $n = 6$).

*Pharmacokinetics*

The plasma concentration time data for each patient were subjected to pharmacokinetic analysis using a computer analysis program (Scaf, 1988). The program includes a correction for infusion time (Loo & Riegelman, 1970) and a statistical analysis comparing the accuracy of models containing different numbers of phases (Boxenbaum *et al.*, 1974). Equations for distribution constants, elimination constants, total body clearance and apparent central and peripheral distribution volumes have been described (Greenblatt & Koch Wester, 1975).

*Statistics*

For statistical analysis of the results Student's $t$ test was used. Differences were considered significant if $P < 0.05$.

### Results

Peak plasma levels of carboplatin were between 20 and 70 mg l$^{-1}$. The fraction present as free Pt in the plasma at the peak level was between 50 and 100%. Either free Pt or free carboplatin were detectable before the second and third

**Figure 2** Carboplatin pharmacokinetics, ○, free platinum; ●, free carboplatin; □, total platinum.

**Table II** Pharmacokinetic parameters (mean ± s.d.) after an i.v. infusion of 250 mg $m^{-2}$ carboplatin given over 30 min at 0, 24 and 48 h

| *Parameter* | *Free Pt* | *Carboplatin* | *Total Pt* |
|---|---|---|---|
| $t_{1/2}\,\alpha$ (min) | 65 ± 30 | 70 ± 22 | 22 ± 15 |
| $t_{1/2}\,\beta$ (min) | 378 ± 156 | 1014 ± 348 | 153 ± 87 |
| $t_{1/2}\,\gamma$ (h) | – | – | 135 ± 53 |
| $Vd_{ss}$ (l $m^{-2}$) | 18 ± 7 | 25 ± 9 | 117 ± 31 |
| AUC (μg $ml^{-1}$ $h^{-1}$) | 36 ± 12 | 74 ± 26 | 174 ± 69 |
| CL TB (ml $min^{-1}$ $m^{-2}$) | 79 ± 23 | 65 ± 27 | 14 ± 6 |

Abbreviations: $Vd_{ss}$, volume of distribution in the steady state; AUC, area under the curve; $Cl_{TB}$, total body clearance.

**Table III** Percent cumulative Pt excretion in the urine after repeated dosing with 250 mg $m^{-2}$ carboplatin i.v. at 0, 24 and 48 h

| *Patient* | *After 6 h* | *24 h* | *48 h* | *72 h* | *144 h* |
|---|---|---|---|---|---|
| 1 | 62.4 | 74.8 | 48.1 | 56.0 | 58.0 |
| 2 | 53.6 | 64.5 | 59.7 | 63.0 | 66.0 |
| 3 | n.d. | 48.5 | n.d. | n.d. | n.d. |
| 4 | 44.0 | 55.8 | 60.7 | 62.0 | n.d. |

n.d. = not done.

infusion, but no longer after the fourth day (more than 24 h after the last infusion).

A representative curve is shown in Figure 2. The plasma pharmacokinetics of carboplatin and free Pt do not differ significantly in any of the parameters from Table II when carboplatin is corrected for the atomic weight of Pt.

The total dose of carboplatin has been calculated by the body surface area. If a correction is applied for creatinine clearance rather larger differences are noticed (Table I). However, between patients 3 and 4 no significant differences could be detected in peak concentrations (10.7, 11.4, 10.2 μg $ml^{-1}$ and 12.4, 14.8, 9.9 μg $ml^{-1}$, respectively) and elimination parameters.

Both a two-compartment and a three-compartment model fit the elimination of free Pt, carboplatin and total Pt. Diversion of the total Pt elimination curve and the other curves becomes apparent after 3–6 h.

The characteristics of the pharmacokinetics of total Pt differ significantly from those of free Pt and carboplatin. $t_{1/2}\,\alpha$ and the total body clearance are shorter ($P < 0.001$), and the area under the curve and the volume of distribution are larger ($P < 0.005$) for total Pt compared to free Pt and carboplatin.

Urinary excretion of free Pt is rapid: in the first 6 h 53% of infused Pt is excreted, and cumulative urinary excretion over a 6 day period is 62% (Table III).

### Discussion

The therapeutic effects of carboplatin closely resemble those of its parent drug cisplatin, especially as far as sensitive tumours are concerned. However, some differences in antitumour activity have been described, such as *in vivo* in metastatic human bladder cancer (MRC Working Party on Urological Cancer, 1987) and *in vitro* in small cell lung cancer (Takahashi *et al.*, 1987).

Such dissimilarities in activity and especially in toxicity may reflect a different effect at the cellular level or in phamacokinetic behaviour of the two compounds. It was demonstrated by Knox *et al.* (1986) that once bound to DNA, the actions of cisplatin and carboplatin were the same. However, DNA lesions occur during incubation *in vitro* after a 110-fold and in cell suspensions after a 45-fold carboplatin dose compared to cisplatin on a molar basis (Micetich *et al.*, 1985). Peak levels of DNA interstrand cross-links occur 6–12 h later in carboplatin treated cells (Micetich *et al.*, 1985). Both phenomena are accounted for by the much slower rate of hydrolysis of carboplatin compared to cis-

**Table IV** Pharmacokinetic parameters after intravenous carboplatin given over 30 min

| *Literature* | $t_{1/2}\,\alpha$ (min) | | | $t_{1/2}\,\beta$ (min) | | | $t_{1/2}\,\gamma$ (days) | $Vd_{ss}$ (l $m^{-2}$) | | | $Cl_{TB}$ (ml $min^{-1}$ $m^{-2}$) | | |
|---|---|---|---|---|---|---|---|---|---|---|---|---|---|
| | *Total Pt* | *Free Pt* | *Carbo-platin* | *Total Pt* | *Free Pt* | *Carbo-platin* | *Total Pt* | *Total Pt* | *Free Pt* | *Carbo-platin* | *Total Pt* | *Free Pt* | *Carbo-platin* |
| Harland *et al.* (1984) 300–500 mg $m^{-2}$ | 102 | 96 | 90 | – | 354 | – | | | | | | | |
| Koeller *et al.* (1986) | | 9.7 | | | 131 | | | | 16 | | | 53.6 | |
| Trump *et al.* (1987) | | | 5.9–35.6 | | | 86–179 | | | | 7.7–10.9 | | | 42–111 |
| Elferink *et al.* (1987) | 22 ± 10 | 23 ± 8 | 16 ± 6 | 116 ± 14 | 120 ± 11 | 118 ± 15 | 4.9 ± 1.5 | 103 ± 32 | 10 ± 1.4 | 9.9 ± 1.3 | | 61 ± 11 | 58 ± 12 |
| Reece *et al.* (1987) 100 mg $m^{-2}$ × 3 days | | | | | | 105 | | | | 17.3 ± 4.2 | | | 105 ± 40 |
| Van Echo *et al.* (1984) 77 mg $m^{-2}$ × 5 days | 26–49 | 7.6–21.4 | | 492–1188 | 102–236 | | | 22.6–69.6 | | | | 40–100 | |
| Newell *et al.* (1987) 800–1600 mg $m^{-2}$ | | 83 ± 15 | | | 366 ± 164 | | | | 20 ± 48 | | | | |

platin. This hydrolysed form is the DNA reactive species. Although it has been shown that cisplatin bound to plasma proteins can, at least in part, still react with strong nucleophilic substances (Hegedüs *et al.*, 1987), protein bound Pt compounds lose most of their cytotoxicity (Gormley *et al.*, 1979). Therefore, the unbound drug or free Pt represents the main source of drug that can be rendered DNA reactive.

The low degree of protein binding in the initial hours following i.v. carboplatin administration is evident from our results and is in agreement with other reports (Harland *et al.*, 1984; Van der Vijgh & Klein, 1986; Elferink *et al.*, 1987). The overlap between elimination curves of the intact carboplatin and free Pt and the lack of statistical differences in pharmacokinetic parameters justify the measurement of free Pt as an indication of the protein binding of carboplatin. Our pharmacokinetic data are congruent with the literature (Table IV) except for the longer elimination half life of carboplatin. The reason for this might be the follow-up of plasma drug concentrations until 96 h, while most studies have a follow-up of 24 h.

The rapid excretion of the drug implies that more than half of the drug dose has left the body in 6 h. The drug is eliminated by the kidney by glomerular filtration and is not secreted by the renal tubular cells (Harland *et al.*, 1984) in contrast to cisplatin. The fast process of reaction with protein-bound sulphydryl groups in the renal tubules, as occurs in cisplatin-induced nephrotoxicity (Weiner & Jacobs, 1983), is therefore unlikely in carboplatin treatment. Even if the drug enters the tubular cells, it leads on a molar level to less DNA toxicity than cisplatin. So, despite an increased renal elimination of carboplatin compared to cisplatin, the nephrotoxicity is much less.

The cumulative urinary excretion after 6 h was in accordance with the literature (Elferink *et al.*, 1987; Harland *et al.*, 1984; Koeller *et al.*, 1986; Smyth *et al.*, 1987), but in patients 1 and 2 it was 20% lower than expected after 6 days (Elferink *et al.*, 1987). The reason for this might be the decreased creatinine clearance and pretreatment with cisplatin (Reece *et al.*, 1986; Mulder *et al.*, 1988).

Correction of carboplatin dose for the renal function has been considered to be of importance (Egorin *et al.*, 1984). In this study differences in total dose corrected for creatinine clearance to a factor of two occurred.

A major object of our study was the determination of a suitable moment for bone marrow reinfusion, crucial to survival of the patients treated in this study. As the main toxic agent, the free carboplatin or the free Pt, is below the detection level in the plasma after 48 h following the last dose, it can be assumed that bone marrow reinfusion can be performed at that moment. The Pt still present at that moment is bound to protein and unlikely to react with bone marrow stem cells. A comparable conclusion is reached by Newell *et al.* (1987) for a single bolus injection.

The authors would like to thank Jan Ymker, Piet Bouma, Henk Bloemhof and Hans Roelevink for drug level measurements.

### References

BOXENBAUM, H.G., RIEGELMAN, S. & ELASHOFF, R.M. (1974). Statistical estimations in pharmacokinetics. *J. Pharmacokinet. Biopharm.*, **2**, 123.

CALVERT, A.H., HARLAND, S.J., NEWELL, D.R. & 9 others (1982). Early clinical studies with *cis*-diammine-1,1 cyclobutane dicarboxylate platinum II. *Cancer Chemother. Pharmacol.*, **9**, 140.

CALVERT, A.H., HARLAND, S.J., NEWELL, D.R., SIDDIK, Z.H. & HARRAP, K.R. (1985). Phase I studies with carboplatin at the Royal Marsden Hospital. *Cancer Treat. Rev.*, **12**, suppl. A, 51.

CANETTA, R., ROZENCWEIG, M. & CARTER, S.K. (1985). Carboplatin: the clinical spectrum to date. *Cancer Treat. Rev.*, **12**, suppl. A, 125.

CURT, G.A., GRYGREL, J.J., CORDEN, B.J. & 5 others (1983). A phase I and pharmacokinetic study of diamminecyclobutanedicarboxylato platinum (NSC 241240). *Cancer Res.*, **43**, 4470.

EGORIN, M.J., VAN ECHO, D.A., TIPPING, Sj. & 4 others (1984). Pharmacokinetics and dosage reduction of *cis*-diammine (1,1 cyclobutane dicarboxylato) platinum in patients with impaired renal function. *Cancer Res.*, **44**, 5432.

ELFERINK, F., VAN DER VIJGH, W.J.F., KLEIN, I., VERMORKEN, J.B., GALL, H.E. & PINEDO, H.M. (1987). Pharmacokinetics of carboplatin after i.v. administration. *Cancer Treat. Rep.*, **71**, 1231.

EVANS, B.D., RAJU, K.S., CALVERT, A.H., HARLAND, Sj. & WILTSHAW, E. (1983). Phase II study of JM8, a new platinum analog, in advanced ovarian carcinoma. *Cancer Treat. Rep.*, **67**, 997.

GORE, M.E., CALVERT, A.H. & SMITH, I.E. (1987). High dose carboplatin in the treatment of lung cancer and mesothelioma: a phase I dose escalation study. *Eur. J. Cancer Clin. Oncol.*, **23**, 1391.

GORMLEY, P.E., BULL, J.M., LEROY, A.F. & CYSYK, R. (1979). Kinetics of *cis-d*-chlorodiammine platinum. *Clin. Pharmacol. Ther.*, **25**, 351.

GREENBLATT, D.J. & KOCH WESTER, J. (1975). Drug therapy. Clinical pharmacokinetics. I. *N. Engl. J. Med.*, **293**, 702.

HARLAND, S.J., NEWELL, D.R., SIDDIK, Z.H., CHADWICK, R., CALVERT, H. & HARRAP, K.R. (1984). Pharmacokinetics of *cis*-diammine-1,1-cyclobutane dicarboxylate platinum (II) in patients with normal and impaired renal function. *Cancer Res.*, **44**, 1693.

HEGEDÜS, L., VAN DER VIJGH, W.J.H., KLEIN, I., KERPEL-FRONIUS, S. & PINEDO, H.M. (1987). Chemical reactivity of cisplatin bound to human plasma proteins. *Cancer Chemother. Pharmacol.*, **20**, 211.

KNOX, R.J., FRIEDLOS, F., LYDALL, D.A. & ROBERTS, J.J. X(1986). Mechanism of cytotoxicity of anticancer platinum drugs: evidence that *cis*-diammine-dichloroplatinum (II) and *cis*-diammine-(1,1-cyclobutanedicarboxylato) platinum (II) differ only in the kinetics of their interaction with DNA. *Cancer Res.*, **46**, 1972.

KOELLER, J.M., TRUMP, D.L., TUTSCH, K.D., EARHART, R.H., DAVIS, T.E. & TORMEY, D.C. (1986). Phase I clinical trial and pharmacokinetics of carboplatin (NSC 241240) by single monthly 30 min infusion. *Cancer*, **57**, 222.

LEROY, A.F., WEHLING, M.L., SPANSELLER, H.L. & 7 others (1977). Analysis of platinum in biological materials by flameless atomic absorption spectrophotometry. *Biochem. Med.*, **18**, 184.

LEYVRAZ, S., OHNUMA, T., LASSUS, M. & HOLLAND, J.F. (1985). Phase I study of carboplatin in patients with advanced cancer, intermittent intravenous bolus, and 24-hour infusion. *J. Clin. Oncol.*, **3**, 1385.

LOO, J.C.K. & RIEGELMAN, S. (1970). Assessment of pharmacokinetic constants from postinfusion blood curves obtained after i.v. infusion. *J. Pharm. Sci.*, **59**, 53.

MEDICAL RESEARCH COUNCIL WORKING PARTY ON UROLOGICAL CANCER, SUBGROUP IN ADVANCED BLADDER CANCER (1987). A phase II study of carboplatin in metastatic transitional cell carcinoma of the bladder. *Eur. J. Cancer Clin. Oncol.*, **23**, 375.

MICETICH, K.C., BARNES, D. & ERICKSON, L.C. (1985). A comparative study of the cytotoxicity and DNA-damaging effects of *cis*-(diammino)(1,1-cyclobutane-dicarboxylato)-platinum (II) and *cis*-diammine dichloroplatinum (II) on L1210 cells. *Cancer Res.*, **45**, 4043.

MULDER, P.O.M., SLEIJFER, D.Th., DE VRIES, E.G.E., UGES, D.R.A. & MULDER, N.H. (1988). Renal dysfunction following high-dose carboplatin treatment. *J. Cancer Res. Clin. Oncol.*, **114**, 212.

NEWELL, D.R., SIDDIK, Z.H., GUMBRELL, L.A. & 4 others (1987). Plasma free platinum pharmacokinetics in patients treated with high dose carboplatin. *Eur. J. Cancer Clin. Oncol.*, **23**, 1399.

OZOLS, R.F., BEHRENS, B.C., OSTCHEGA, Y. & YOUNG, R.C. (1985). High dose cisplatin and high dose carboplatin in refractory ovarian cancer. *Cancer Treat. Rev.*, **12**, suppl. A, 59.

REECE, P.A., STAFFORD, I., RUSSELL, J. & GILL, P.G. (1986). Reduced ability to clear ultrafilterable platinum with repeated courses of cisplatin. *J. Clin. Oncol.*, **4**, 1392.

REECE, P.A., BISHOP, J.F., OLVER, I.N., STAFFORD, I., HILLCOAT, B.L. & MORSTYN, G. (1987). Pharmacokinetics of unchanged carboplatin (CBDCA) in patients with small cell lung carcinoma. *Cancer Chemother. Pharmacol.*, **19**, 326.

ROSE, W.C. & SCHURIG, J.E. (1985). Preclinical antitumor and toxicologic profile of carboplatin. *Cancer Treat. Rev.*, **12**, suppl. A, 1.

ROZENCWEIG, M., NICAISE, C., BEER, M. & 4 others (1983). Phase I study of carboplatin given on a five day intravenous schedule. *J. Clin. Oncol.*, **1**, 621.

SCAF, A.H.J. (1988). Pharmacokinetic analysis with rugfit; an interactive pharmacokinetic computer program. *Biopharm. Drug Disp.*, **9**, 415.

SIDDIK, Z.H., NEWELL, R., JONES, M. & BOXALL, F.E. (1982). Pharmacokinetics of *cis*-diammine-1,1-cyclobutane dicarboxylate platinum (II) (CBDCA JM8) in mice and rats. *Proc. Am. Assoc. Cancer Res.*, **23**, 168.

SMYTH, R.D., OGURI, S., SAKAKIBARA, T. & 4 others (1987). Clinical pharmacokinetics of carboplatin. *J. Clin. Pharmacol.*, **27**, 716.

TAKAHASHI, H., SASAKI, Y., SAIJO, N. & 6 others (1987). *In vitro* colony inhibition of carboplatin against stomach and lung cancer cell lines in comparison with cisplatin. *Cancer Chemother. Pharmacol.*, **19**, 197.

TRUMP, D.L., GREM, J.L., TUTSCH, K.D. & 5 others (1987). Platinum analogue combination chemotherapy: cisplatin and carboplatin – a phase I trial with pharmacokinetic assessment of the effect of cisplatin administration on carboplatin excretion. *J. Clin. Oncol.*, **5**, 1281.

VAN DER VIJGH, W.J.F. & KLEIN, I. (1986). Protein binding of five platinum compounds. *Cancer Chemother. Pharmacol.*, **18**, 129.

VAN ECHO, D.A., EGORIN, M.J., WHITACRE, M.Y., OLMAN, E.A. & AISNER, J. (1984). Phase I clinical and pharmacologic trial of carboplatin daily for 5 days. *Cancer Treat. Rep.*, **68**, 1103.

WEINER, M.W. & JACOBS, C. (1983). Mechanism of cisplatin nephrotoxicity. *Fed. Proc. Fed. Am. Soc. Exp. Biol.*, **42**, 2974.

WILTSHAW, E. (1985). Ovarian trials at the Royal Marsden. *Cancer Treat. Rev.*, **12**, suppl. A, 67.